# Exosomal EphA2 Promotes Gastric Cancer Progression by Inducing Phenotypic Transformation of Tumor Cells in a Ligand-Independent Manner

**DOI:** 10.3390/cells15141253

**Published:** 2026-07-12

**Authors:** Linan Zhan, Rui Wang, Furong Liu, Wanming Li, Jie Zhang, Xuetong Zhou, Wei Ba, Shuo Chen, Jin Fang

**Affiliations:** 1 Department of Cell Biology, Key Laboratory of Cell Biology, Ministry of Public Health, Key Laboratory of Medical Cell Biology, Ministry of Education, China Medical University, Shenyang 110122, China; 2 Department of Sports Physiology and Biochemistry Teaching and Research Section, Shenyang Sport University, Shenyang 110102, China; 3 Department of Stem Cells and Regenerative Medicine, Basic Medical Sciences of China Medical University, Shenyang 110122, China

**Keywords:** tumor heterogeneity, exosomal EphA2, gastric cancer, ligand-independent manner

## Abstract

The heterogeneity of tumor cells facilitates their dynamic adaptation to tumor microenvironmental pressures throughout progression. Nevertheless, the mechanisms underlying intercellular communication and transformation among heterogeneous tumor cells remain inadequately understood. In this study, we indicate that Ephrin type-A receptor 2 (EphA2) is heterogeneously expressed in gastric cancer (GC) tumor cells, with those exhibiting elevated EphA2 (EphA2^High^) expression demonstrating enhanced migratory and invasive capabilities. EphA2^High^ cells facilitate the transfer of EphA2 via exosomes, which subsequently localize on the membrane of EphA2^Low^ cells, thereby activating the ERK signaling pathway in a ligand-independent manner. This process promotes the transformation of EphA2^Low^ cells and contributes to the progression of GC. An investigation into the correlation between serum levels and tumor metastasis in patients with GC revealed that those with lymph node metastasis exhibited higher levels of serum exosomal EphA2. This study elucidates the process of dominant group formation within heterogeneous tumor cells and suggests the viability of exosomal EphA2 as a potential biomarker for further clinical investigation.

## 1. Introduction

Gastric cancer (GC) is the fifth most common malignant tumor and the fourth leading cause of cancer-associated death worldwide [1,2]. Notably, most patients with GC present with metastatic disease at the start of the diagnostic process [3]. Clarifying the mechanisms underlying tumor metastasis is of paramount importance for the clinical management of patients with GC. Accumulated evidence has revealed that the heterogeneity of GC cells is closely related to tumor metastasis [4,5,6]. Tumor heterogeneity has been demonstrated to exist in a dynamic state, adapting to the demands of rapid tumor progression [7,8]. Within these heterogeneous tumor cells, those exhibiting more aggressive and malignant behaviors can induce the transformation of less malignant cells, eventually becoming the dominant cell type and thereby facilitating tumor progression [9]. Nonetheless, the transformation modes and the underlying potential mechanisms remain inadequately understood.

Tumor heterogeneity adapts to the demands of rapid tumor progression through heterogeneous tumor cells’ intercellular signaling [7,10], cell–cell adhesion [11], and physical force [12]. Among these, exosomes are spherical vesicles ranging from 30 to 150 nm in diameter and possessing a bilayer membrane structure that plays a crucial role in this process. Exosomes can be secreted by a variety of cell types, including immune cells, mesenchymal stem cells, epithelial cells, astrocytes, endothelial cells, and tumor cells, encapsulating proteins, lipids, DNA, and RNA [13,14,15]. Consequently, exosomes carry significant biological information from tumor cells and may serve as important mediators of heterogeneous tumor cells’ intercellular substance transport. Shang et al. [16] found that exosomal circPACRGL derived from colorectal cancer cells with high expression of circPACRGL, compared to those with low expression, significantly promoted the oncogenic role in colorectal cancer cell proliferation and metastasis via the miR-142-3p/miR-506-3p-TGFβ1 axis. Xu et al. [17] demonstrated that exosome-mediated intercellular transformation of CXCR1^Low^ to CXCR1^High^ tumor cells promotes the progression of head and neck squamous cell carcinoma. Sun et al. [18] found that exosomal miR-335-5p derived from metastatic colorectal cancer cells promotes non-metastatic colorectal cancer cell invasion and metastasis by facilitating EMT via targeting RASA1.

The Ephrin type-A receptor 2 (EphA2), a member of the receptor tyrosine kinase family, is markedly overexpressed in various tumor types and is implicated in the process of tumor cell proliferation, migration, invasion, angiogenesis, and epithelial–mesenchymal transition [19,20]. Furthermore, elevated expression of EphA2 is associated with poor prognosis, enhanced metastatic potential, and reduced survival rates in tumor patients [21,22]. EphA2 facilitates various biological activities during tumor development via exosomal pathways. Gan et al. [23] identified that exosomal EphA2 promoted M2-type polarization of macrophages by promoting activation of the PI3K/AKT/mTOR pathway in macrophages, thereby promoting the progression of renal cell carcinoma. Gao et al. [24] found that the increase in EphA2 in exosomes derived from drug-resistant cells may be an important mechanism for the progression of breast cancer induced by chemotherapy. Liu et al. [25] showed that the EphA2 is enriched in exosomes of triple-negative breast cancer, which can break the endothelial monolayer barrier by downregulating tight junction proteins of endothelial cells, providing a new mechanism for exosomal EphA2 to promote breast cancer metastasis. However, whether exosomal EphA2 delivery influences tumor progression in GC has not yet been reported.

Under normal circumstances, EphA2 interacts with Ephrin-A1 (EFNA1) in adjacent cells and induces various signal networks after cell-to-cell contact. EFNA1, as a membrane protein, participates in the forward (Ephrin: EphA2) and reverse (EphA2: Ephrin) signal transduction, which is also called ligand-receptor bidirectional signal transduction [20]. We investigated here that the introduction of exosomes enriched with EphA2 regulates EphA2^Low^ cell function and occurs through a ligand-independent pathway of EphA2, rather than through the interaction between EphA2 and its ligand EFNA1, which is a new discovery for the mechanism of action of exosomal EphA2 in GC heterogeneity.

Our study demonstrates that the expression of EphA2 in GC cells is heterogeneous. Tumor cells exhibiting elevated EphA2 expression possess enhanced migratory and invasive capabilities. EphA2^High^ cells disseminate EphA2 via exosomes, which subsequently localize on the membranes of EphA2^Low^ cells. This process activates the ERK signaling pathway in EphA2^Low^ cells in a ligand-independent manner, thereby facilitating their transformation and contributing to the progression of GC. An investigation into the correlation between serum exosomal EphA2 levels and tumor metastasis in patients with GC revealed that those with lymph node metastasis exhibited higher levels of serum exosomal EphA2. This study offers novel insights and a molecular foundation into the dynamic alterations within heterogeneous tumor cells and the clinical diagnosis and targeting of GC.

## 2. Methods

### 2.1. Patient and Specimen Collection

In this study, whole blood samples were obtained from Shengjing Hospital of China Medical University. All patients provided written informed consent. This study was approved by the Ethics Review Committee of Chinese Medical University (ethical code is [2016]029). We collected 4 mL of whole blood from GC patients, centrifuged it at 1000 rpm for 10 min at 4 °C, and collected the supernatant to obtain the serum.

### 2.2. Cell Culture

Human GC cell lines SGC7901, MGC803, HGC27, and BGC823 were obtained from the Typical Culture Preservation Commission Cell Bank, Chinese Academy of Sciences (Shanghai, China), and were cultured at 37 °C in a 5% CO_2_ atmosphere. SGC7901, MGC803, and BGC823 were cultured in high-glucose DMEM medium, and HGC27 was cultured in RMIP 1640 medium. All media were supplemented with 10% fetal bovine serum (Sangon Biotech, Shanghai, China) and 1% penicillin–streptomycin (Solarbio, Beijing, China, P1400). Mycoplasma contamination was excluded using the Myco-Lumi™ Luminescent Mycoplasma Detection Kit (Beyotime, Shanghai, China, C0298), with B/A ratios below the threshold of 0.9 for both cell lines.

### 2.3. Western Blot

After extracting the total protein from each cell line and exosomes, the protein was quantified by a BCA kit (Beyotime, Shanghai, China, P0012), followed by SDS-PAGE electrophoresis, membrane transfer, blocking, primary antibody incubation, TBST washing, and secondary antibody incubation, then imaging in a chemiluminescence instrument (Tanon, Shanghai, China). The sources of antibodies were: EphA2 (Santa Cruz, sc-398832), GAPDH (ZSGB-BIO, Beijing, China, TA-08), Alix (Invitrogen, Waltham, MA, USA, MA1-83977), CD9 (Invitrogen, Bothell, WA, USA, 10626D), CD63 (Invitrogen, Bothell, WA, USA, 10628D), ERK (Bioss, Beijing, China, bsm-2637R), p-ERK (Zenbio, Chengdu, China, R24245), AKT (Immunoway, Suzhou, China, YT0185), p-AKT (Cell Signaling Technology, Waltham, MA, USA, 31957), Flag (ABclonal, Wuhan, China, AE005), EFNA1 (ABclonal, Wuhan, China, A9132), p-EphA2-S897 (ABclonal, Wuhan, China, AP1082), p-EphA2-Y772 (ABclonal, Wuhan, China, AP0817). CD9, ALIX, and calnexin were used as exosomal markers. GAPDH (ZSGB-BIO, Beijing, China, TA-08) was used as a loading control.

### 2.4. Lentivirus Transfection

In this study, shRNA-EphA2(5′-GCGCCUGUUCACCAAGAUUTT-3′, 3′-TTCGCGGACAAGUGGUUCUAA-5′) and shRNA-Ctrl lentiviral vectors were purchased from Sangon Biotech (Shanghai, China); overexpression-EphA2 and overexpression-Ctrl lentiviral vectors were purchased from Genechem (Shanghai, China).

SGC7901 cells and MGC803 cells were inoculated into six-well plates with 1 × 10^5^ cells per well, and the knockdown or overexpression lentivirus was transfected using the pro-transfection reagent polybrene after 12 h. Their respective negative control lentiviruses were also transfected at the same time. After transfection for 48 h, puromycin was used for screening, and after screening for 72 h, the knockdown or overexpression level of EphA2 was identified by Western blot.

### 2.5. CCK8 Assay

MGC803 cells were collected after 24 h of co-incubation with exosomes, counted, and inoculated into 96-well plates with 2 × 10^3^ cells per well. 10 μL CCK8 reagent (Beyotime, C0038) was added to each well after 24 h of incubation, and the absorbance value at 450 nm was measured after 1 h of incubation again. The above operation was repeated once a day, and the proliferation curve was plotted according to the absorbance value after 5 days.

### 2.6. Clonogenic Assay

The above cells were seeded into 6-well plates, 1 × 10^3^ cells per well. After 2 weeks of culture, the cells were fixed with methanol, stained with 0.1% crystal violet, washed with PBS, and dried at room temperature. The number of clones was counted and analyzed statistically.

### 2.7. Transwell Assay

After co-incubation with exosomes, the cells were inoculated into the transwell chamber of 24-well plates. For the migration assay, 2 × 10^4^ cells were added directly to the upper chamber, and for the invasion, cells were inoculated after adding matrix gel (Corning, NY, USA, 354234) to the upper chamber. The upper chamber was incubated with 200 μL FBS-free medium, and the lower chamber was incubated with 600 μL complete medium. After 24 h of incubation, the chambers were removed, fixed using methanol, stained with crystal violet, and photographed to count the number of cells on the bottom surface of the membrane.

### 2.8. Animal Studies

All mice used in this study were 4-week-old female BALB/c nude mice obtained from Charles River (Beijing, China). All animal experiments were approved by the Institutional Animal Care and Use Committee of China Medical University (protocol code CMU2020048). A total of 51 female BALB/c nude mice were used across all experiments. Mice were randomly divided into groups using a computer-generated random number sequence, and the investigator was blinded to group allocation during tumor measurement and data analysis.

Specifically, for the subcutaneous tumor formation model, MGC803 cells co-incubated with oeCtrl- or oeEphA2-derived exosomes were injected into the left and right axillae of nude mice (3 mice in total). Subcutaneous tumor formation in BALB/c mice injected with shCtrl-SGC7901 or shEphA2-SGC7901 cells was performed with *n* = 5 per group (10 mice in total). In addition, subcutaneous tumor formation in BALB/c mice injected with oeCtrl-MGC803 cells (*n* = 5) or oeEphA2-MGC803 cells (*n* = 4) involved a total of 9 mice. A suspension of MGC803, shCtrl-SGC7901, shEphA2-SGC7901, oeCtrl-MGC803, or oeEphA2-MGC803 cells (4 × 10^6^ cells in 100 μL PBS) was injected into each mouse. Tumor tissues were harvested after 4 weeks, weighed, and measured for volume calculation.

For the metastatic tumor model, shCtrl-SGC7901, shEphA2-SGC7901, oeCtrl-MGC803, or oeEphA2-MGC803 cells (1 × 10^6^ cells in 100 μL PBS) were injected into nude mice via the tail vein, with *n* = 5 per group (20 mice in total). Lungs and livers were collected 4 weeks post-injection and examined for metastatic nodules.

In vivo imaging to detect metastatic signals was performed with *n* = 3 per group for PBS, oeCtrl-exo, and oeEphA2-exo (9 mice in total). A stable luciferase-expressing MGC803-Luc cell line was established by transfecting MGC803 cells with a luciferase-encoding lentivirus (Genechem, Shanghai, China). To establish a metastatic microenvironment, nude mice were injected via the tail vein with PBS, oeCtrl-MGC803 exosomes, or oeEphA2-MGC803 exosomes every other day for four consecutive injections. On the day following the fourth injection, MGC803-Luc cells (1 × 10^6^ cells in 100 μL PBS) were injected via the tail vein, and exosome administration was resumed every other day for eight additional injections. The mice were monitored regularly and maintained under standard conditions. After 70 days, the mice were anesthetized, and upon loss of the righting reflex, D-luciferin potassium salt (Beyotime, ST196) was administered intraperitoneally. In vivo imaging was performed 15 min later using a small-animal imaging system (Bruker, Billerica, MA, USA). The mice were then euthanized, and lung and liver tissues were harvested for ex vivo imaging to confirm metastatic loci.

### 2.9. Immunohistochemistry

Tumor tissues harvested from nude mice were fixed in 4% paraformaldehyde. Tissue embedding and sectioning were outsourced to Baihao Biotech (Shenyang, China). The procedure was performed following the immunohistochemistry protocol (MXB, KIT-9710). EphA2 primary antibody was diluted 1:100 and applied to tissue sections, followed by overnight incubation at 4 °C in a humidified chamber. The next day, a biotin-conjugated secondary antibody was applied and incubated for 30 min at room temperature. DAB chromogenic solution (MXB, Beijing, China, DAB-2031) and hematoxylin counterstain were then applied sequentially. After dehydration and clearing, slides were mounted and imaged under brightfield microscopy.

### 2.10. H&E Staining

Tissue sections were processed using the H&E staining kit (Solarbio, Beijing, China, G1120). Following deparaffinization and rehydration, sections were stained with hematoxylin, differentiated, counterstained with eosin, dehydrated, cleared, and cover-slipped. Images were acquired using a digital slide scanner.

### 2.11. Exosome Isolation

Exosomes were isolated using differential ultracentrifugation. First, fetal bovine serum (FBS) was ultracentrifuged at 150,000× *g* for 6 h to deplete exosomes, generating exosome-free medium. Cells were cultured in this exosome-free medium for 48 h prior to collection. Conditioned medium was then subjected to differential ultracentrifugation: supernatants were sequentially centrifuged at 300× *g* for 10 min, 2000× *g* for 20 min, and 10,000× *g* for 30 min to remove cellular debris. The final exosome pellet was obtained by ultracentrifugation at 150,000× *g* for 3 h.

For human serum exosome isolation, an Exosome Isolation Kit (Invitrogen, 4478360) was used according to the manufacturer’s protocol. Serum samples were incubated with extraction reagent at 4 °C overnight, followed by centrifugation at 1500× *g* for 30 min. The resulting exosome pellet was resuspended in PBS and stored at −80 °C.

### 2.12. Exosome Identification

The characteristic markers of exosomes were identified by Western blot. The morphology of exosomes was identified by transmission electron microscopy (HITACHI, Tokyo, Japan). The copper mesh coated with carbon film was placed in the exosome droplets and incubated at room temperature for 15 min. Then, the copper mesh was placed in a 2% tungsten phosphate dye solution for dyeing for 30 s. The copper mesh was removed and dried in air for 15 min. Then, the imaging was performed under transmission electron microscopy. The size and concentration analysis of exosomes was commissioned by XP Biomed (Shanghai, China).

### 2.13. Immunofluorescence

The exosomes were incubated with DID (Invitrogen, WA, USA, V22887) at a ratio of 1000:1 for 30 min, then the exosomes were washed with PBS, and then the exosomes were co-incubated with MGC803 cells for 24 h. The cells were fixed using 4% paraformaldehyde; DAPI (Beyotime, Shanghai, China, C1002) was used for nuclear staining, PBS for washing, and imaged under confocal microscopy (Nikon, Tokyo, Japan).

In contrast, exosomes with Flag labeling were directly co-incubated with MGC803 cells for 24 h. After incubation, they were sequentially blocked, incubated with Flag primary antibody, Alexa594 fluorescent secondary antibody (Invitrogen, A32740), DAPI for nuclear staining, and finally imaged under confocal microscopy.

### 2.14. siRNA Transfection

The transfection of siRNA (Sangon Biotech, Shanghai, China) was carried out in a 12-well plate according to the instructions of the RNA transfection kit (Ribo, Suzhou, China, C10511-05), and the transfection efficiency was verified by Western blot after 24 h of transfection.

The sequence of siRNA-EFNA1 is as follows:

siEFNA1-1: 5′-CCAUGACAAUCCACAGGAGAA TT-3′

3′-UUCUCCUGUGGAUUGUCAUGGTT-5′

siEFNA1-2: 5′-CGUCUUCUGGAACAGUUCAAA TT-3′

3′-UUUGAACUGUUCCAGAAGACGTT-5′

siEFNA1-3: 5′-CCGCACUAUGAAGAUCACUCUTT-3′

3′-AGAGUGAUCUUCAUAGUGCGGTT-5′

siRNA-NC: 5′-UUCUCCGAACGUGUCACGUTT-3′

3′-ACGUGACACGUUCGGAGAA TT-5′

The sequence of siRNA-ERK is as follows:

siERK-1: 5′-AAGUUCGAGUAGCUAUCA A-3′

3′-UUGAUAGCUACUCGAACUU-5′

siERK-2: 5′-GGACCUCAUGGAAACAGAU-3′

3′-AUCUGUUUCCAUGAGGUCC-5′

siERK-3: 5′-GCAUGGUGUGCUCUGCUUA-3′

3′-UAAGCAGAGCACACCAUGC-5′

siERK-NC: 5′-UUCUCCGAACGUGUCACGUTT-3′

3′-ACGUGACACGUUCGGAGAATT-5′

### 2.15. ELISA

The extracted serum exosomes were added to the 96-well ELISA plate and performed according to the ELISA kit (Jianglai, Shanghai, China, JL13552) instructions. In the end, the absorbance of each well was read at 450 nm.

### 2.16. Statistical Analysis

All statistical analyses and graph plotting were performed using GraphPad Prism 7.0 software. Data were presented as the mean ± SD. Student’s *t*-test (two-sided) was used to compare two groups. One-way ANOVA and two-way ANOVA were used to determine multiple group comparisons, followed by Tukey’s honest significant difference post hoc test. The patient’s OS was analyzed using Kaplan–Meier analysis. *p* < 0.05 was considered statistically significant.

## 3. Result

### 3.1. The Function Heterogeneity of EphA2 in GC Cells Is Related to Tumor Progression

**Figure 1:**
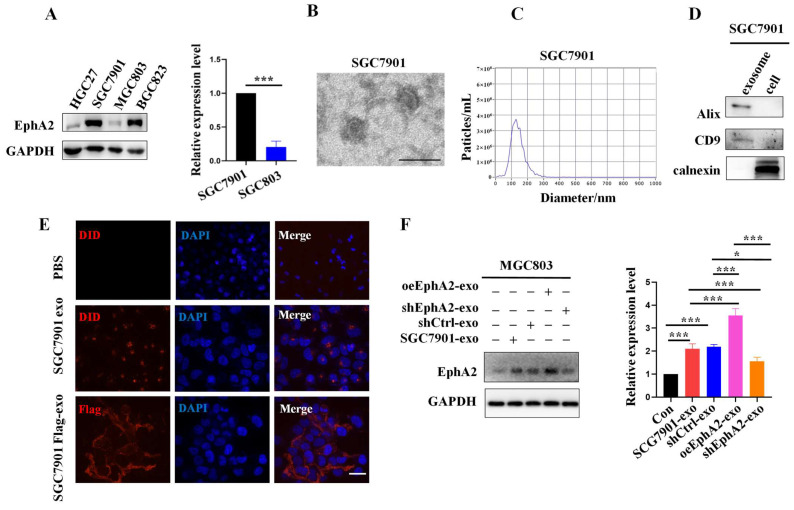
**EphA2 derived from EphA2^High^ cell exosomes can be internalized by EphA2^Low^ cells.** (**A**) Expression levels of EphA2 in four GC cell lines, *n* = 3. (**B**) TEM characterization of exosome morphology (scale bar = 100 nm). (**C**) Particle size distribution of exosomes analyzed by NTA. (**D**) Western blot detection of exosomal markers (positive: Alix and CD9; negative: calnexin) in SGC7901 cells and exosomes. (**E**) Localization of exogenous exosomal EphA2 in MGC803 (scale bar = 25 μm). (**F**) Expression levels of EphA2 in MGC803 cells following treatment with exosomes derived from different cell sources, *n* = 3. (* *p* < 0.05, *** *p* < 0.001).

GC is a highly heterogeneous tumor; we detected the expression of EphA2 in four GC cell lines (Figure 1A) and found that the expression of EphA2 was relatively high in SGC7901 and BGC823 cell lines but relatively low in MGC803 and HGC27 cell lines. Here, we chose the EphA2^High^ cell line SGC7901 and the EphA2^Low^ cell line MGC803 as research objects. To explore the function of EphA2 in GC cells, we knocked down EphA2 in the EphA2^High^ cell line SGC7901 and overexpressed EphA2 in the EphA2^Low^ cell line MGC803 by lentivirus transfection and verified the transfection efficiency by Western blot (Figure S1A,B). The effect of EphA2 on the proliferation of GC cells was detected using CCK8 and clone formation experiments. The results showed that when EphA2 was knocked down, the proliferation ability of the EphA2^High^ cells decreased, while after EphA2 was overexpressed, the proliferation ability of the EphA2^Low^ cells increased significantly (Figure S1C–F), suggesting that EphA2^High^ cells had stronger proliferation ability than EphA2^Low^ cells in vitro. Next, we detected the influence of EphA2 on the migration and invasion of GC cells using a transwell assay. When EphA2 was knocked down, the migration and invasion ability of the EphA2^High^ cells decreased, while after EphA2 was overexpressed, the migration and invasion ability of the EphA2^Low^ cells increased significantly (Figure S1G,H), which indicated that EphA2^High^ cells had stronger migration and invasion abilities than EphA2^Low^ cells in vitro.

We further explore these through in vivo experiments. ShCtrl SGC7901, shEphA2 SGC7901, oeCtrl MGC803 cells, and oeEphA2 MGC803 cells were injected subcutaneously into the armpit of BALB/c mice, and the diameter of the tumor was measured every week for four weeks. After four weeks, the mice were euthanized, the tumor was removed and weighed, and the volume was calculated. The results showed that among the mice injected with shCtrl SGC7901 cells and shEphA2 SGC7901 cells, the tumor tissue in the shCtrl group continued to grow with increasing injection time, while no tumor was formed in the shEphA2 group (Figure S2A–D). Among the mice injected with oeCtrl MGC803 and oeEphA2 MCG803 cells, only 50% of the mice in the oeCtrl group had tumors, and the tumor volume and weight of the mice injected with oeEphA2 MCG803 cells were significantly higher than those of oeCtrl MGC803 cells (Figure S2E–H). These results indicate that EphA2^High^ cells also have stronger proliferation ability than EphA2^Low^ cells in vivo. To further explore the influence of EphA2 on GC cell metastasis in vivo, we injected shCtrl SGC7901, shEphA2 SGC7901, oeCtrl MGC803, and oeEphA2 MGC803 cells into BALB/c mice via tail vein injection. After four weeks, the mice were euthanized, the organs of the mice were observed, and it was found that there were two cases of lung metastasis in mice injected with shCtrl SGC7901 cells, but in mice injected with shEphA2 SGC7901 cells, there was no metastasis, which was further confirmed by HE staining (Figure S2I). However, the mice injected with oeCtrl MGC803 cells did not show any metastasis, while the mice injected with oeEphA2 MGC803 cells had one lung metastasis and one liver metastasis (Figure S2J). Taken together, these observations suggest that EphA2^High^ cells tend to exhibit more active malignant behavior than EphA2^Low^ cells, and the functional heterogeneity of EphA2 in GC cells may be associated with tumor progression.

### 3.2. EphA2 Derived from EphA2^High^ Cell Exosomes Can Be Internalized by EphA2^Low^ Cells

We suspect that the interaction pattern between GC heterogeneous tumor cells may be related to the exosomes. Therefore, we extracted the exosomes from the EphA2^High^ cell SGC7901. By transmission electron microscopy (TEM), we observed that the SGC7901 exosomes had a typical double-layer membrane structure and were disk-shaped (Figure 1B). According to Nanoparticle Tracking Analysis (NTA), 98.6% of SGC7901 exosomes were concentrated in the range of 30–150 nm (Figure 1C). Western blot analysis confirmed the presence of exosomal markers (positive: Alix and CD9; negative: calnexin) in SGC7901 cells and exosomes (Figure 1D), indicating that the exosomes of SGC7901 cells without cell contamination were successfully extracted. We also transfected Flag–EphA2 into EphA2^High^ SGC7901 cells and determined the transfection efficiency by Western blot (Figure S3A).

We then determined whether exosomal EphA2 from EphA2^High^ cells could be internalized into the EphA2^Low^ cells. We treated EphA2^Low^ cells with exosomes from EphA2^High^ cells incubated with DiD dye or transfected with Flag-tagged EphA2 for 12 h and observed the internalization and localization of the exosomes in the EphA2^Low^ cells under a confocal microscope. After co-incubation, obvious red fluorescence was observed in the EphA2^Low^ cells, which were located on the cell membrane (Figure 1E). The same result was confirmed by Western blot (Figure S3B). The expression of EphA2 in recipient cells was subsequently detected. The exosomes of oeEphA2 SGC7901 and shEphA2 SGC7901 cells were extracted, and the transfection efficiency was determined (Figure S3C,D). We added oeEphA2 SGC7901 cell-derived exosomes and shEphA2 SGC7901 cell-derived exosomes to EphA2^Low^ cells. The expression of EphA2 in recipient cells also changed consistently with different exosomes (Figure 1F). These results indicate that exosomal EphA2 can be internalized into recipient cells.

### 3.3. Exosomal EphA2 Derived from EphA2^High^ Cells Transforms EphA2^Low^ Cells to Promote GC Progression In Vitro

**Figure 2:**
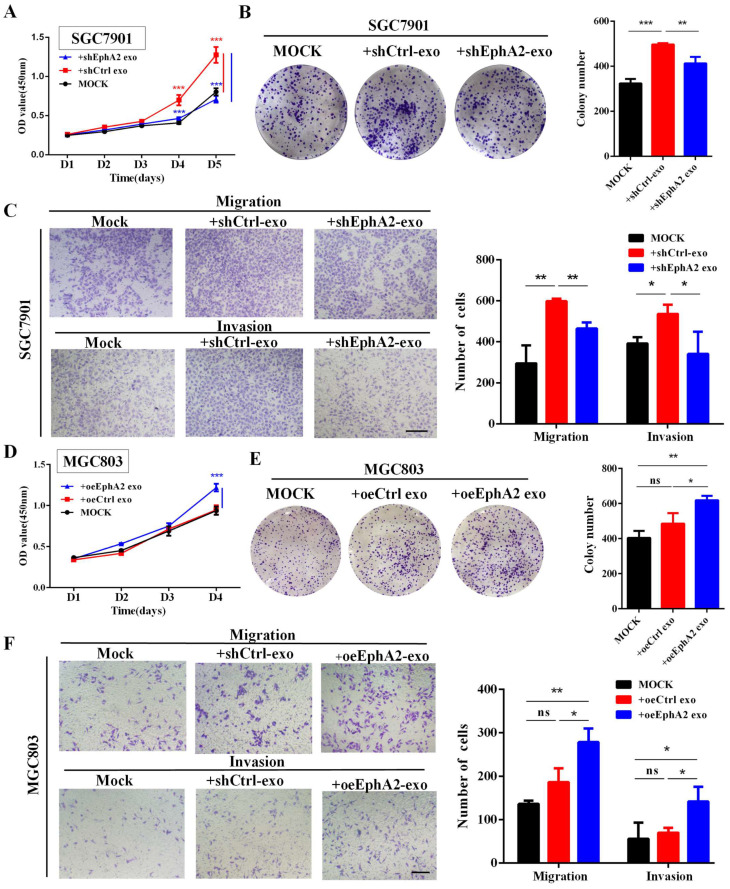
**Exosomal EphA2 derived from EphA2^High^ cells transforms EphA2^Low^ cells to promote GC progression in vitro.** (**A**) Proliferation capacity of SGC7901 cells following treatment with shCtrl- or shEphA2-derived exosomes, analyzed by CCK8 assay, *n* = 3. (**B**) Proliferation capacity of SGC7901 cells following treatment with shCtrl- or shEphA2-derived exosomes, detected by colony formation assay, *n* = 3. (**C**) Migration and invasion abilities of SGC7901 cells following treatment with shCtrl- or shEphA2-derived exosomes, detected by transwell assay, *n* = 3 (scale bar = 200 μm). (**D**) Proliferation capacity of MGC803 cells following treatment with shCtrl- or oeEphA2-derived exosomes, analyzed by CCK8 assay, *n* = 3. (**E**) Proliferation capacity of MGC803 cells following treatment with shCtrl- or oeEphA2-derived exosomes, detected by colony formation assay, *n* = 3. (**F**) Migration and invasion abilities of MGC803 cells following treatment with shCtrl- or oeEphA2-derived exosomes, detected by transwell assay, *n* = 3 (scale bar = 200 μm). (ns, not significant, * *p* < 0.05, ** *p* < 0.01, *** *p* < 0.001).

To investigate whether exosomal EphA2 from EphA2^High^ cells induces transformation in EphA2^Low^ cells to facilitate GC progression, we performed knockdown of EphA2 in EphA2^High^ SGC7901 cells. Then, shCtrl SGC7901 and shEphA2 SGC7901 exosomes were added to EphA2^Low^ cells. Through CCK8 and clone formation experiments, it was found that compared with the shCtrl SGC7901 exosome group, the proliferation and clone formation ability of EphA2^Low^ cells were obviously weakened after the addition of shEphA2 SGC7901 exosomes (Figure 2A,B), indicating that exosomal EphA2 can promote the proliferation of EphA2^Low^ cells. We then examined exosomal EphA2 on the metastatic ability of EphA2^Low^ cells. It was found that the migration and invasion abilities of EphA2^Low^ cells in the shEphA2 SGC7901 cell group were significantly reduced compared to the shCtrl SGC7901 exosome group (Figure 2C).

We further overexpressed EphA2 in EphA2^Low^ MGC803 cells. Then, we extracted and characterized exosomes from MGC803 cells. TEM revealed that the exosomes had a typical disk shape (Figure S4A). The particle size distribution of 96% of the exosomes of MGC803 was concentrated in the range of 30–150 nm (Figure S4B). Western blot analysis confirmed the presence of exosomal markers (positive: Alix and CD9; negative: calnexin) in MGC803 cells and exosomes (Figure S4C). The above results showed that we successfully extracted exosomes from MGC803 cells without causing cell pollution. EphA2 was overexpressed in MGC803 cells by lentivirus transfection, and the transfection efficiency was verified by Western blot (Figure S4D). We then added oeCtrl MGC803 and oeEphA2 MGC803 cell-derived exosomes to MGC803 cells. Through CCK8, clone formation, and transwell assays, it was found that the proliferation, clone formation, migration, and invasion abilities of MGC803 cells were enhanced in the oeEphA2 MGC803 exosome group compared with the oeCtrl MGC803 exosome group (Figure 2D–F). The results showed that exosomal EphA2-mediated transformation of EphA2^Low^ to EphA2^High^ cells in vitro.

### 3.4. Exosomal EphA2 Derived from EphA2^High^ Cells Transforms EphA2^Low^ Cells to Promote GC Progression In Vivo

**Figure 3:**
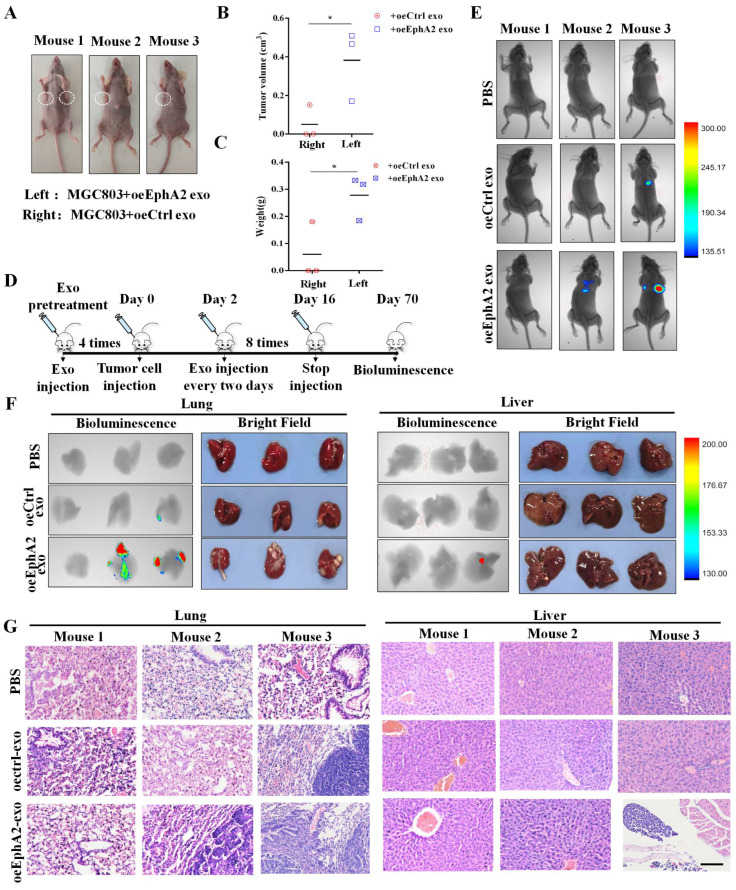
**Exosomal EphA2 derived from EphA2^High^ cells transforms EphA2^Low^ cells to promote GC progression in vivo.** (**A**) Subcutaneous tumor formation in the left and right axillae of nude mice after injection with oeCtrl- or oeEphA2-derived exosomes (*n* = 3 mice). (**B**) Comparison of tumor volume between the two groups. (**C**) Comparison of tumor weight between the two groups. (**D**) Schematic diagram of the experimental design for tail-vein injection of oeCtrl- or oeEphA2-derived exosomes in nude mice (*n* = 3 mice). (**E**) In vivo imaging to detect metastatic signals in nude mice. (**F**) In vivo imaging to detect visceral metastatic foci in nude mice. (**G**) H&E staining to detect the formation of lung and liver metastatic foci after injection with oeCtrl- or oeEphA2-derived exosomes (scale bar = 100 μm). (* *p* < 0.05).

To determine whether exosomal EphA2-mediated transformation of EphA2^Low^ to EphA2^High^ cells promotes GC progression in vivo, we performed the following experiments. We co-incubated exosomes of oeCtrl MGC803 and oeEphA2 MGC803 cells with MGC803 cells, collected the co-incubated cells, and subcutaneously injected the two groups of cells into the left and right armpits of nude mice to observe tumor formation. The results showed that after co-incubation with oeEphA2 MGC803 cell exosomes, the tumorigenicity of MGC803 cells was significantly higher than that of the control group (Figure 3A), and the volume and weight of the tumors were significantly increased compared to those of the control group (Figure 3B,C), indicating that exosomal EphA2 can promote the proliferation of EphA2^Low^ cells in vivo. We then evaluated the effect of exosomal EphA2 on the metastatic ability of EphA2^Low^ cells in vivo. We administered exosomes derived from oeCtrl MGC803 and oeEphA2 MGC803 cells into BABL/c mice through the tail vein for pretreatment and created a metastatic microenvironment in vivo. The PBS group was set as a blank control, which was injected once every other day for a total of four administrations. Following the fourth injection, luciferase-labeled MGC803 cells were introduced via tail vein injection, marking this point as day 0. On the second day, oeCtrl MGC803 exosomes, oeEphA2 MGC803 exosomes, and PBS were injected again, once every other day for eight times, and on the 70th day, in vivo imaging was performed (Figure 3D). Mice injected with oeCtrl MGC803 and oeEphA2 MGC803 exosomes showed metastasis signals, but mice injected with oeEphA2 MGC803 exosomes had a high fluorescence signal ratio and a large fluorescence area, whereas mice injected with PBS did not show any fluorescence signal expression (Figure 3E). To further clarify the expression site of the fluorescence signal, we removed the organs of the mice after euthanasia and imaged them again. Mice injected with oeEphA2 MGC803 exosomes not only had lung metastasis but also liver metastasis. Mice injected with oeCtrl MGC803 exosomes only had lung metastasis, whereas mice injected with PBS did not show any metastasis (Figure 3F). All the above experiments were identified by HE staining (Figure 3G). These results indicated a trend towards increased metastatic burden in the oeEphA2-exo group compared to controls, providing supportive evidence for the pro-metastatic role of exosomal EphA2.

### 3.5. Exosomal EphA2 Derived from EphA2^High^ Cells Transforms EphA2^Low^ Cells to Promote GC Progression Through Ligand-Independent Pathways

EphA2 leads to two-way signal transduction between contact cells by binding to its ligand, EFNA1. In addition, EphA2 can regulate biological function in tumor cells through ligand-independent pathways [26,27].

**Figure 4:**
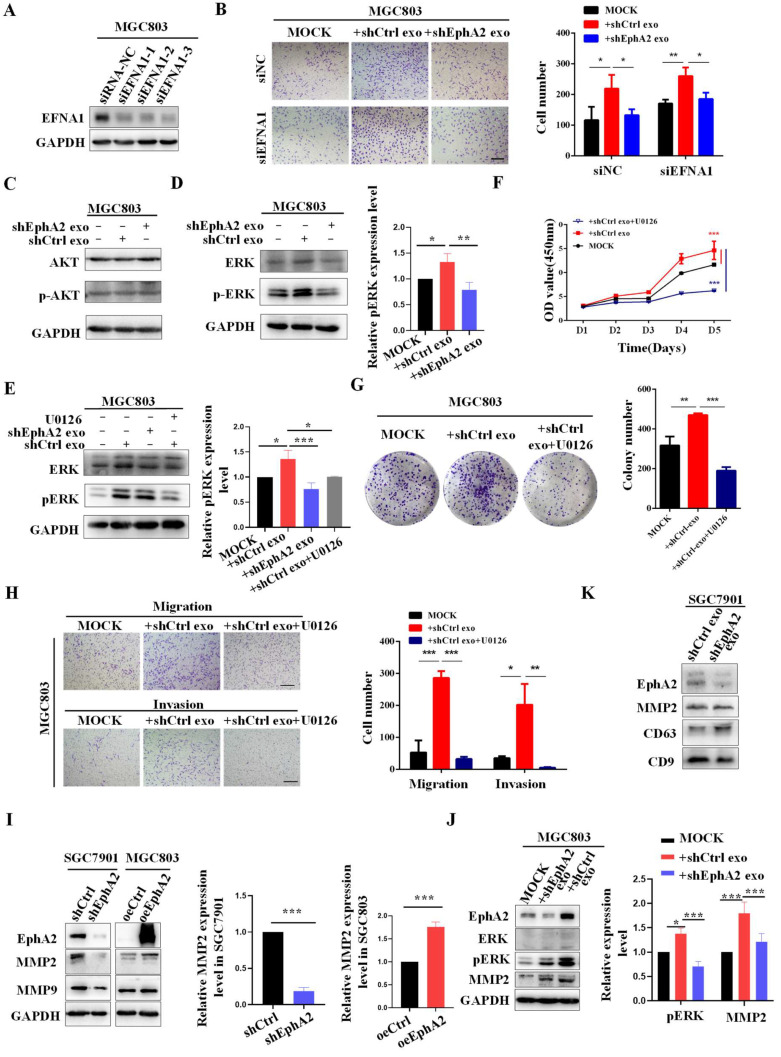
**Exosomal EphA2-mediated transformation of EphA2^Low^ to EphA2^High^ cells promotes GC progression through ligand-independent pathways.** (**A**) Western blot analysis of EFNA1 silencing after siRNA transfection. (**B**) Transwell assay to detect changes in the migration ability of MGC803 cells after the addition of shCtrl or shEphA2 exosomes in the context of EFNA1 silencing, *n* = 3 (scale bar = 200 μm). (**C**) Western blot detection of the impact of exosome addition on the AKT signaling pathway. (**D**) Effect of exosome addition on the ERK signaling pathway, *n* = 3. (**E**) Expression of p-ERK after the addition of the ERK inhibitor U0126, *n* = 3. (**F**) CCK8 assay to assess the proliferation ability of MGC803 cells treated with U0126 in the presence of exosomes, *n* = 3. (**G**) Colony formation assay to evaluate the colony-forming ability of MGC803 cells treated with U0126 in the presence of exosomes, *n* = 3. (**H**) Transwell assay to measure the migration and invasion abilities of MGC803 cells treated with U0126 in the presence of exosomes, *n* = 3 (scale bar = 200 μm). (**I**) Expression levels of MMP2 and MMP9 in SGC7901 and MGC803 cells after EphA2 knockdown or overexpression, *n* = 3. (**J**) Expression levels of p-ERK and MMP2 in MGC803 cells after exosome treatment, *n* = 3. (**K**) Expression level of MMP2 in SGC7901 exosomes after EphA2 knockdown. (* *p* < 0.05, ** *p* < 0.01, *** *p* < 0.001).

In this study, EphA2 entered recipient cells in the form of exosomes and caused changes in the function of EphA2^Low^ cells. We first confirmed whether exosomal EphA2 binds to EFNA1 on the surface of EphA2^Low^ cells, thus activating the reverse signal of EphA2-EFNA1 to regulate cell function. We transfected siRNA into EphA2^Low^ cells to silence the expression of EFNA1. All three sequences achieved efficient knockdown at the protein level, and we selected siEFNA1-1 as the representative sequence for subsequent functional assays. The transfection efficiency was verified by Western blot (Figure 4A). Next, exosomes from shCtrl SGC7901 and shEphA2 SGC7901 cells were added to EphA2-low cells in which EFNA1 had been silenced. Functional changes in the recipient cells were then assessed by colony formation assay (Figure S5A), Transwell assay (Figure 4B), and by measuring the expression of pERK and MMP2 (Figure S5B). After EFNA1 silencing, the proliferation, migration, invasion, and levels of pERK and MMP2 in EphA2-low cells treated with shCtrl SGC7901 exosomes remained significantly higher than those in cells treated with shEphA2 SGC7901 exosomes. This indicates that exosomal EphA2 does not rely on EFNA1 to modulate recipient cell functions. Therefore, these findings confirm that exosomal EphA2 drives the conversion of EphA2^Low^ cells to an EphA2^high^ phenotype, thereby promoting GC progression via a ligand-independent pathway.

Studies have confirmed that the function of EphA2 is mainly related to the PI3K/AKT and ERK signaling pathways [28,29]. Western blot showed that the expression of pAKT did not change (Figure 4C); however, the expression of pERK was altered (Figure 4D) after the exosomes of shEphA2 SGC7901 cells were added to the recipient cells, which preliminarily confirmed that exosomal EphA2 regulated the function of recipient cells by activating the ERK signaling pathway.

To further verify the involvement of the ERK signaling pathway, we co-treated EphA2^Low^ cells with shCtrl SGC7901 exosomes and the ERK phosphorylation inhibitor U0126. Western blot analysis revealed that pERK levels remained suppressed, similar to the effect observed with shEphA2 SGC7901 exosomes (Figure 4E). To assess the functional implications of this mechanism, we treated EphA2^Low^ cells with shCtrl SGC7901 exosomes in combination with U0126. CCK8 and colony formation assays demonstrated that SGC7901 exosomes did not enhance the proliferation capacity of EphA2^Low^ cells upon the addition of U0126, suggesting that exosomal EphA2 modulates the proliferation of recipient cells through activation of the ERK signaling pathway (Figure 4F,G). Similarly, transwell assays showed that SGC7901 exosomes did not promote the migration or invasion abilities of EphA2^Low^ cells after the addition of U0126, indicating that exosomal EphA2 regulates the metastatic capacity of recipient cells via the ERK signaling pathway (Figure 4H).

To complement the pharmacological inhibition approach, we performed genetic knockdown of ERK using siRNA. The knockdown efficiency was confirmed by Western blot (Figure S6A). We then treated MGC803 cells with SGC7901 exosomes under siRNA-NC or siERK-3 silencing conditions. Consistent with the U0126 results, CCK8 assays showed that ERK knockdown abrogated the exosome-induced enhancement of proliferation (Figure S6B), and transwell assays demonstrated that the exosome-promoted migration and invasion were similarly abolished upon ERK silencing (Figure S6C). Collectively, these findings from both pharmacological and genetic approaches confirm that exosomal EphA2 exerts its regulatory effects on recipient cells through activation of the ERK signaling pathway.

MMP2 and MMP9 are the classic downstream molecules of EphA2 [30]. To further detect the effector molecules downstream of the ERK pathway, MMP2 and MMP9 in shEphA2 SGC7901 cells and oeEphA2 MCG803 cells were detected using Western blot. The results showed that when EphA2 was knocked down or overexpressed, compared with MMP9, the expression of MMP2 significantly decreased or increased (Figure 4I). To further verify whether the expression of MMP2 was affected by exosomal EphA2, exosomes of shEphA2 SGC7901 cells were added to the EphA2^Low^ cells, and it was found that the expression of MMP2 also changed with pERK level (Figure 4J). We further detected the expression level of MMP2 in the exosomes of shEphA2 SGC7901 cells and found that the expression of MMP2 in the exosomes was not affected by the knockdown of EphA2 (Figure 4K). These results indicate that exosomal EphA2 regulates MMP2 in EphA2^Low^ cells by activating the ERK signaling pathway.

### 3.6. Correlation Between EphA2 Expression in Serum Exosomes of Patients with GC and Tumor Metastasis

**Figure 5:**
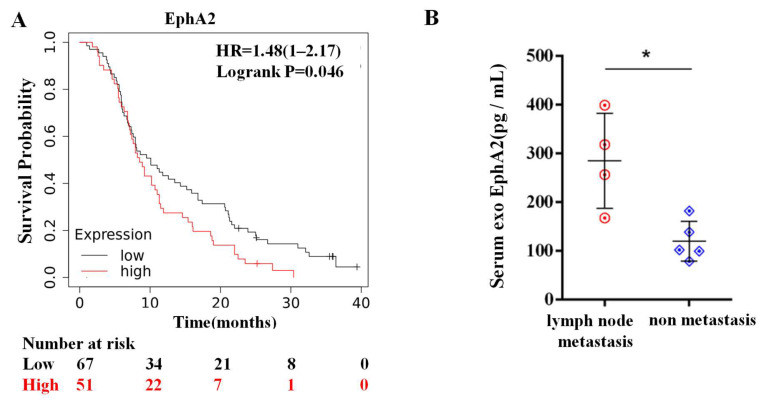
**Correlation between EphA2 expression in serum exosomes of patients with GC and tumor metastasis.** (**A**) Overall survival of GC patients with high EphA2 expression (*n* = 118). (**B**) Detection of EphA2 expression in human serum exosomes by ELISA (*n* = 9). (* *p* < 0.05).

To understand the relationship between the expression of EphA2 in GC cells and tumor progression, we analyzed the relationship between the total survival time and the expression of EphA2 in 118 GC patients using the Kaplan–Meier Plotter database. Kaplan–Meier analysis using the Kaplan–Meier Plotter database (*n* = 118) showed a trend towards shorter overall survival in patients with high EphA2 expression (HR 1.48, *p* = 0.046) (Figure 5A), providing supportive evidence for a potential association between EphA2 expression and GC progression.

To prove the correlation between the expression of EphA2 in serum exosomes of GC patients and tumor metastasis, we detected EphA2 in serum exosomes of nine GC patients by ELISA. The results indicated that some patients with GC had relatively high levels, whereas those in others were relatively low. We then analyzed the patient’s medical records and found that all patients with relatively high expression levels of EphA2 in serum exosomes had lymph node metastasis, while others had no metastasis (Figure 5B). Therefore, we speculated that serum exosomal EphA2 levels in GC patients correlate with tumor metastasis. Given the small cohort size (*n* = 9), these results will require further validation.

## 4. Discussion

With the swift advancements in single-cell transcriptome analysis, the significance of tumor cell heterogeneity in tumor progression has been acknowledged [31,32]. Nonetheless, the communication mechanisms among heterogeneous tumor cells remain incompletely understood. Here, we present a transformation mode to elucidate the impact of EphA2^High^ cells, characterized by enhanced migratory and invasive capabilities, on the malignant behavior of EphA2^Low^ cells, thereby facilitating the progression of GC.

Exosomes, which serve as mediators of intercellular communication, have been extensively documented to influence tumor metastasis by inducing metabolic reprogramming, immune evasion, and drug resistance [33,34,35]. This study demonstrates that exosomes can facilitate tumor progression by activating inactive GC tumor cell populations. Our findings underscore the significant role of exosomes in modulating dynamic alterations within heterogeneous tumor cell populations. The content of exosomes is meticulously regulated by their originating cells, making the diverse composition of exosomes from heterogeneous host cells crucial for the transmission of regulatory information. We have identified for the first time that exosomes derived from EphA2^High^ GC cells can modulate the malignant behavior of EphA2^Low^ cells via exosomal EphA2, exerting a significant regulatory influence on tumor progression.

EphA2 belongs to the receptor tyrosine kinase family, has a molecular weight of 130 kDa, and consists of 976 amino acid residues, including the extracellular N-terminal domain, transmembrane domain, and intracellular C-terminal domain. The extracellular domain includes an LBD binding domain, an EGF-like domain, and two fibronectin III domains, while the intracellular domain includes the tyrosine kinase domain, SAM domain, and PDZ binding motif [22,36]. Moreover, EphA2 is positively correlated with increased tumor invasion depth, advanced TNM staging, and lymph node metastasis [19,20]. This study demonstrates that cells with high EphA2 expression exhibit more aggressive malignant behavior compared to those with low EphA2 expression, underscoring the critical role of heterogeneous EphA2 expression in tumor progression. Consequently, identifying effective strategies to inhibit the malignant transformation driven by EphA2^High^ cells within heterogeneous tumors may offer a precise therapeutic target for GC treatment. Our research demonstrates that exosomal EphA2 from EphA2^High^ cells plays a critical role in this process, and there is currently no report about the function of exosomal EphA2 in GC. To investigate the role of exosomal EphA2 in GC, we knocked down EphA2 in EphA2^High^ cells and overexpressed EphA2 in EphA2^Low^ cells. Subsequently, we incubated the exosomes from EphA2 knockdown and overexpression cells with EphA2^Low^ cells. The findings indicated that upregulation of EphA2 in exosomes led to significant enhancement in the proliferation, clonal formation, migration, and invasion capabilities of EphA2^Low^ cells. Conversely, downregulation of EphA2 in exosomes resulted in a marked reduction in the proliferation, migration, and invasion potential of EphA2^Low^ cells. Subsequent in vivo experiments corroborated these results, demonstrating that the overexpression of exosomal EphA2 significantly augmented the metastatic potential of EphA2^Low^ cells to the lungs and liver. The findings suggest that exosomal EphA2 influences the malignant behavior of EphA2^Low^ cells, ultimately facilitating the progression of GC.

We further examined the molecular mechanisms through which exosomal EphA2 facilitates GC progression. Upon secretion, exosomes can transfer their signaling molecules to recipient cells via at least three distinct mechanisms: endocytosis, membrane fusion, and receptor-ligand interaction, with endocytosis being the predominant mode of exosome uptake [37]. In various recipient cells, exosomes can be internalized through endocytosis mediated by reticulin, caveolin, and lipid rafts. Upon endocytosis, exosomes may either fuse with endosomes or be directed to lysosomes for degradation. Furthermore, the exosomal membrane can merge with the plasma membrane, facilitating the delivery of exosomal contents into the recipient cell. Alternatively, exosomes can interact with homologous receptors on the recipient cell membrane, thereby initiating intracellular signaling cascades [37,38]. Exosomes were extracted from SGC7901 cells transfected with a Flag–EphA2 and subsequently incubated with MGC803 cells. Immunofluorescence experiments revealed that exosomes containing Flag–EphA2 predominantly localized to the cell membrane of the recipient cells. This observation led us to hypothesize that exosomes with EphA2 may preferentially fuse with recipient cells or modulate their function through ligand-receptor interactions. To date, no research has specifically addressed the localization of exosomal EphA2 within recipient cells. Our study provides the first confirmation of this phenomenon and proposes that elucidating the binding mechanism of exosomal EphA2 with recipient cells may enhance our comprehension of the molecular mechanisms underlying exosomal EphA2.

EphA2 regulates the physiological function of cells through the activation of its natural ligand EFNA1, but studies show that EphA2 plays a more complicated role in tumors. Under normal circumstances, EphA2 interacts with EFNA1 in adjacent cells and induces various signal networks after cell-to-cell contact. EFNA1, as a membrane protein, participates in forward (Ephrin: EphA2) and reverse (EphA2: Ephrin) signal transduction, which is also called ligand-receptor bidirectional signal transduction. The forward signal promotes the oligomerization and phosphorylation of EphA2, thus enhancing the kinase activity, while the reverse signal transduction is more likely to be non-kinase-dependent due to the lack of enzyme activity of EFNA1 [39,40,41]. We next investigated whether exosomal EphA2 activates the ligand-dependent reverse signaling pathway by binding to its ligand, EFNA1. Given that exosomal EphA2 is localized on the receptor cell membrane, we further explored the pathway activation. Our findings indicate that the concentration of EphA2 within exosomes can also influence the functionality of recipient cells, as silencing EFNA1, the EphA2-EFNA1 reverse signaling pathway, is not activated. We hypothesize that the introduction of exosomes enriched with EphA2 regulates recipient cell function through a ligand-independent pathway of EphA2, rather than through the interaction between EphA2 and its ligand EFNA1. The findings partially diverge from the existing report. Gao et al. [42] confirmed that exosomal EphA2 from drug-resistant cancer cells activates the ERK signaling pathway through the EphA2–Ephrin A1 reverse signaling pathway, thus promoting the metastasis of breast cancer cells. Takasugi et al. [24] confirmed that exosomes secreted by aging cells can promote the proliferation of cancer cells through EphA2, and this process is also realized by activating the ERK signaling pathway through a ligand-dependent reverse signaling pathway. Yamashita et al. [43] found that exosomal EphA2 derived from lung cancer cells also promotes endothelial cell angiogenesis through a ligand-dependent reverse signaling pathway. Furthermore, Hong et al. [44] proposed that ligand-independent activation of EphA2 is initiated by VEGF released from cancer-associated fibroblast-conditioned medium, thereby contributing to GC tumorigenesis. Notably, the study did not find evidence of EphA2 being secreted in exosomal form or from GC tumor cells. Our study is a novel finding for the mechanism of action of EphA2 in GC heterogeneity.

**Figure 6:**
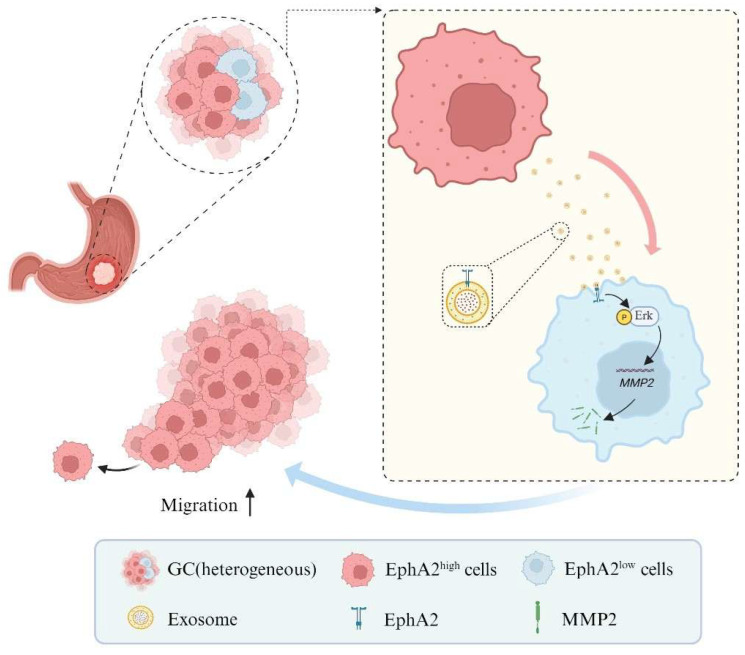
Schematic model illustrating how exosomal EphA2 derived from EphA2^High^ cells transforms EphA2^Low^ cells to promote gastric cancer progression in a ligand-independent manner. The figure was Created in BioRender. Zhan, L. (2026) https://BioRender.com/lsuww4f.

In conclusion, our study demonstrated that EphA2^High^ cells disseminate EphA2 via exosomes, facilitating its localization on the membrane of EphA2^Low^ cells. This process activates the ERK signaling pathway in EphA2^Low^ cells in a ligand-independent manner, thereby promoting the transformation of EphA2^Low^ cells and subsequently contributing to the progression of GC (Figure 6). A preliminary analysis into the association between serum exosomal EphA2 levels and metastasis in GC patients revealed that patients with lymph node metastasis exhibited elevated serum exosomal EphA2 expression compared to those with non-metastatic GC. This finding preliminarily suggests that exosomal EphA2 may hold potential relevance for diagnosis and treatment, although this remains to be validated in larger cohorts. The study’s outcomes not only elucidate the interaction between heterogeneous GC tumor cells and their impact on tumor progression but also offer novel insights into potential molecular mechanisms. Overall, our findings suggest that exosomal EphA2 could represent a candidate diagnostic and therapeutic target for GC.

## Data Availability

The original contributions presented in this study are included in the article/Supplementary Material. Further inquiries can be directed to the corresponding author.

## Supplementary Materials

The following supporting information can be downloaded at https://www.mdpi.com/article/10.3390/cells15141253/s1, Figure S1: The function heterogeneity of EphA2 in GC cells is related to tumor progression in vitro; Figure S2: The function heterogeneity of EphA2 in GC cells is related to tumor progression in vivo; Figure S3: EphA2 derived from EphA2^High^ cell exosomes can be internalized by EphA2^Low^ cells; Figure S4: Identification of exosome derived from MGC803 cells; Figure S5: Functional changes in MGC803 cells following treatment with shCtrl or shEphA2 exosomes under EFNA1 silencing; Figure S6: Functional changes in MGC803 cells following treatment with SGC7901-derived exosomes under siRNA-NC or siERK knockdown; Table S1: List of abbreviations; Table S2: Baseline clinical and pathological features of the study cohort (*n* = 9).
