# Supplementary material for: Exosomal EphA2 Promotes Gastric Cancer Progression by Inducing Phenotypic Transformation of Tumor Cells in a Ligand-Independent Manner
Source: Cells. 2026 Jul 12;15(14):1253. doi: 10.3390/cells15141253 (PMC13406668; doi:10.3390/cells15141253)
Supplement: Supplementary file 1 [file cells-15-01253-s001.zip › cells-4312221-supplementary.pdf]

Table S1. List of abbreviations

Abbreviation	Full name
EphA2	Ephrin type-A receptor 2
EFNA1	Ephrin-A1
GC	Gastric cancer
Exo	Exosomes
shRNA	Short hairpin RNA
siRNA	Small interfering RNA
CCK8	Cell Counting Kit-8
pERK	Phosphorylated extracellular signal-regulated kinase
MMP2	Matrix metalloproteinase 2
U0126	MEK1/2 inhibitor
DiD	1,1'-dioctadecyl-3,3,3',3'-tetramethylindodicarbocyanine
IF	Immunofluorescence
HE	Hematoxylin and eosin
DAPI	4',6-diamidino-2-phenylindole
NTA	Nanoparticle Tracking Analysis
oeCtrl	Overexpression control
oeEphA2	Overexpression of EphA2
shCtrl	Short hairpin RNA control
shEphA2	Short hairpin RNA targeting EphA2
siEFNA1	Small interfering RNA targeting EFNA1
siNC	Small interfering RNA negative control

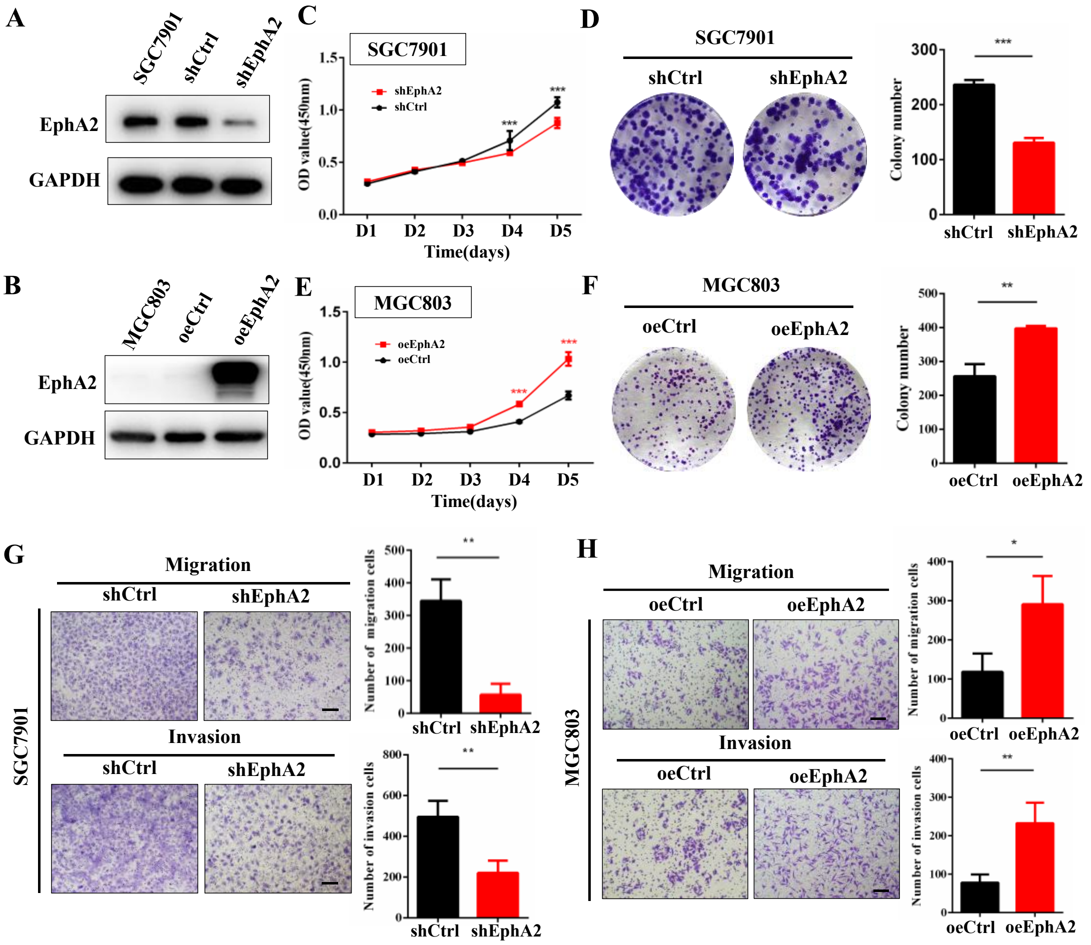

Figure S1. The function heterogeneity of EphA2 in GC cells is related to tumor progression in vitro. A. EphA2 expression in SGC7901 cells transfected with shCtrl or shEphA2 by western blot. **B.** EphA2 expression in MGC803 cells transfected with oeCtrl or oeEphA2 by western blot. **C.** Proliferation capacity of SGC7901 cells after EphA2 knockdown by CCK8. **D**. Proliferation capacity of SGC7901 cells after EphA2 knockdown by colony formation assays. **E.** Proliferation capacity of MGC803 cells after EphA2 overexpression by CCK8. **F.** Proliferation capacity of MGC803 cells after EphA2 overexpression by colony formation assays. **G.** Migration and invasion abilities of SGC7901 cells following EphA2 knockdown by transwell (Scale bar = 200 μm). **H.** Migration and invasion abilities of MGC803 cells following EphA2 overexpression by transwell (Scale bar = 200 μm). (* $p < 0.05$, ** $p < 0.01$, *** $p < 0.001$).

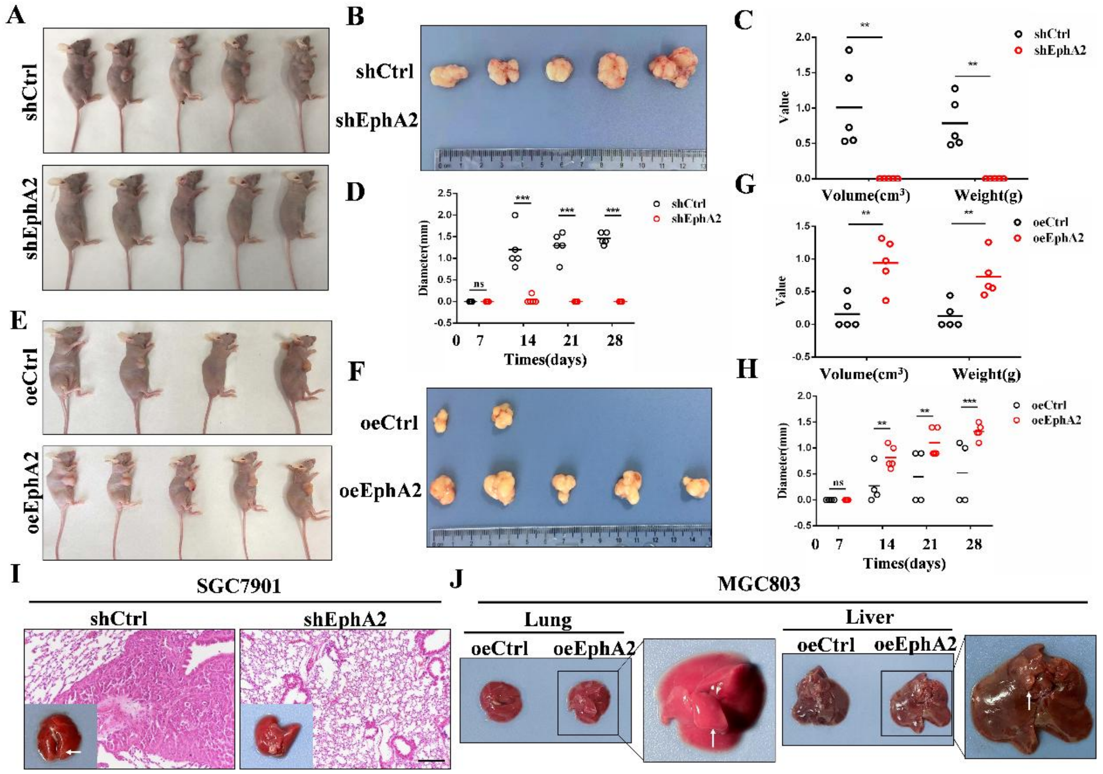

Figure S2. The function heterogeneity of EphA2 in GC cells is related to tumor progression in vivo. A. Subcutaneous tumor formation in BALB/c mice injected with shCtrl-SGC7901 or shEphA2-SGC7901 cells (n=5 mice). **B–D.** Effects of EphA2 knockdown on tumor size in nude mice, including time-dependent changes in tumor diameter, and comparison of tumor volume and weight. **E.** Subcutaneous tumor formation in BALB/c mice injected with oeCtrl-MGC803 (n=5 mice) or oeEphA2-MGC803 cells (n=4 mice). **F–H.** Effects of EphA2 overexpression on tumor size in nude mice, including time-dependent changes in tumor diameter, and comparison of tumor volume and weight. **I.** H&E staining to detect lung metastasis foci in mice after tail-vein injection of shCtrl-SGC7901 or shEphA2-SGC7901 cells (n=5 mice) (Scale bar = 200 μm). **J.** Detection of lung and liver metastases in mice injected with oeCtrl-MGC803 or oeEphA2-MGC803 cells using stereomicroscopy (n=5 mice). (**$p < 0.01$, ***$p < 0.001$).

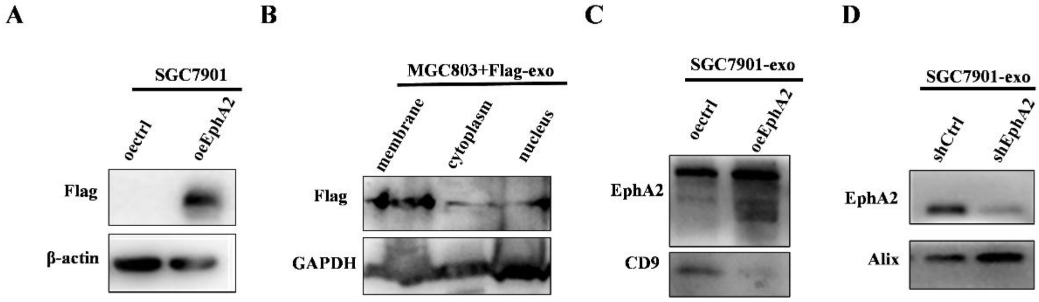

Figure S3. EphA2 derived from EphA2^{High} cell exosomes can be internalized by EphA2^{Low} cells. A. Western blot analysis of the Flag-EphA2 expression in SGC7901 cells after EphA2-Flag overexpression. **B**. Western blot analysis of the Flag-EphA2 expression in the cell membrane of MGC803 cell after treated with EphA2-Flag overexpression exosome. **C.** Western blot analysis of

the EphA2 expression in SGC7901-derived exosome after overexpression. **D.** Western blot analysis of the EphA2 expression in SGC7901-derived exosome after siRNA transfection.

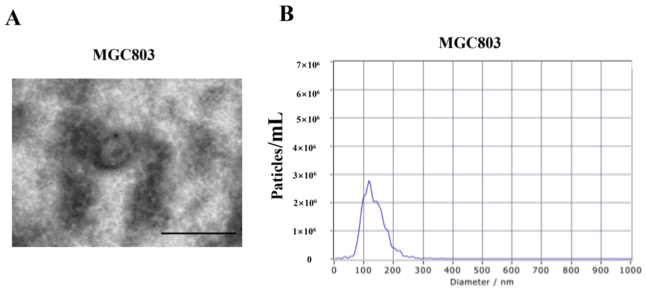

Figure S4. Identification of exosome derived from MGC803 cells. A. TEM characterization of exosome morphology (Scale bar = 100 nm). **B**. Particle size distribution of exosomes analyzed by NTA. **C**. Western blot detection of exosomal markers (positive: Alix and CD9; negative: calnexin) in MGC803 cells and exosomes. **D.** Western blot analysis of the EphA2 expression in MGC803-derived exosome after EphA2 overexpression.

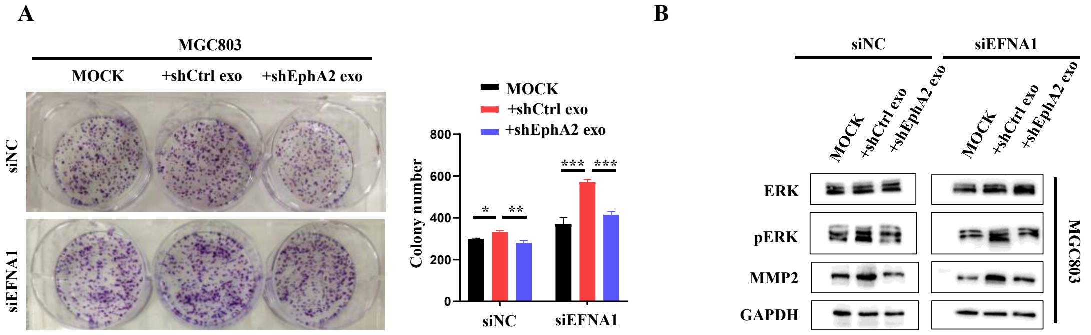

Figure S5. Functional changes of MGC803 cells following treatment with shCtrl or shEphA2 exosomes under EFNA1 silencing. A. Colony formation assay to detect changes in the proliferation ability of MGC803 cells after the addition of shCtrl or shEphA2 exosomes in the context of EFNA1 silencing, *n*=3. **B**. Western blot assay to detect expression level of pERK and MMP2 in MGC803 cells after the addition of shCtrl or shEphA2 exosomes in the context of EFNA1 silencing. (*** $p < 0.001$).

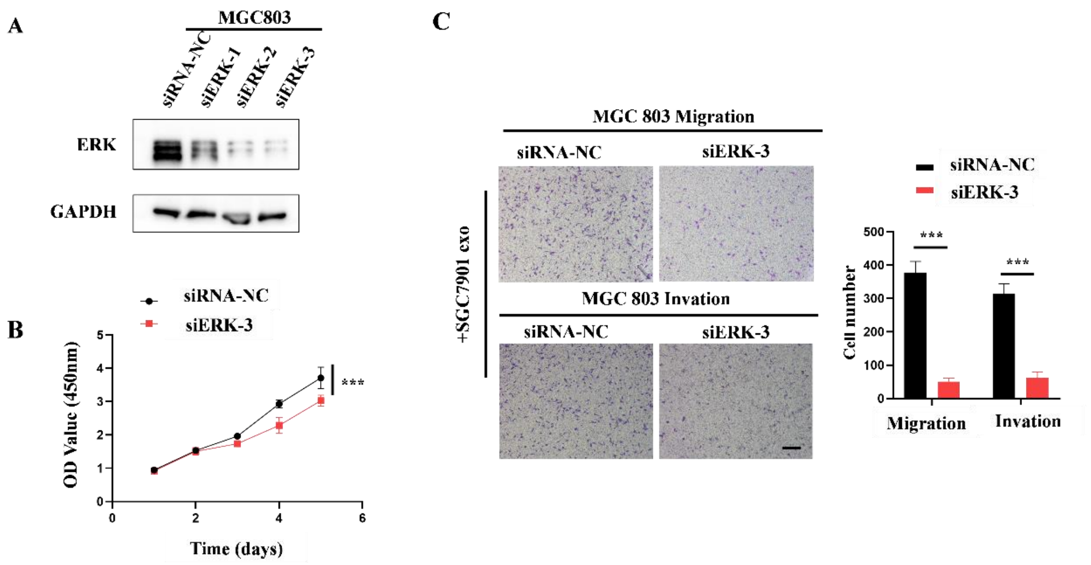

Figure S6. Functional changes in MGC803 cells following treatment with SGC7901-derived exosomes under siRNA-NC or siERK knockdown. A. Western blot analysis of ERK silencing after siRNA transfection. **B.** CCK8 assay to detect changes in the proliferation ability of MGC803 cells after the addition of SGC7901 exosomes in the context of siRNA-NC or siERK-3 knockdown, n=3. **C**. Transwell assay to measure the migration and invasion abilities of MGC803 cells treated with SGC7901 exosomes in the context of siRNA-NC or siERK-3 knockdown, n=3 (Scale bar = 200 μm). (*** $p < 0.001$).

Table S2. Baseline clinical and pathological features of the study cohort (n=9).

Patient ID	Gender	Age	Blood type	Pathological diagnosis	Lauren type	Pathol-ogical T stage	Lymph node status
1	male	66	A	Gastric ulcer with intramucosal adenocarcinoma, poorly differentiated, invading the submucosa	intestinal type	T3	No metastasis
2	female	66	A	Gastric adenocarcinoma, poorly differentiated, invading the full thickness of the gastric wall	intestinal type	T3	metastasis
3	female	45	O	Gastric adenocarcinoma, poorly differentiated, invading the full thickness of the gastric wall	Diffuse type	T3	metastasis
4	male	73	O	Gastric carcinoma with lymphoid stroma, invading the deep muscular layer near the serosa	intestinal type	T2	No metastasis
5	male	60	A	Gastric carcinoma with lymphoid stroma, invading the	Mixed type	T2	No metastasis

				deep muscular layer near the serosa			
6	female	43	B	Gastric mucosal adenocarcinoma, poorly differentiated, invading the full thickness	Diffuse type	T4	metastasis
7	male	56	B	Gastric adenocarcinoma, poorly differentiated, invading the subserosa	intestinal type	T3	No metastasis
8	male	53	O	Gastric adenocarcinoma, poorly differentiated, invading the full thickness	intestinal type	T3	metastasis
9	female	71	A	Gastric adenocarcinoma, moderately to poorly differentiated, invading the serosal adipose tissue	intestinal type	T2	No metastasis
